# Current and future requirements to industrial analytical infrastructure—part 1: process analytical laboratories

**DOI:** 10.1007/s00216-020-02420-2

**Published:** 2020-02-15

**Authors:** Kristina Eisen, Tobias Eifert, Christoph Herwig, Michael Maiwald

**Affiliations:** 1 Arbeitskreis Prozessanalytik, Gesellschaft Deutscher Chemiker, 60486 Frankfurt am Main, Germany; 2 Daiichi Sankyo Europe GmbH, 81379 Munich, Germany; 3 Covestro Deutschland AG, 47829 Krefeld/Uerdingen, Germany; 4 ICEBE, Research Area Biochemical Engineering, TU Wien, 1060 Vienna, Austria; 5 Bundesanstalt für Materialforschung und -prüfung (BAM), 12489 Berlin, Germany

**Keywords:** Smart test laboratories, Laboratory 4.0, Sustainable production, Industry 4.0

## Abstract

The competitiveness of the chemical and pharmaceutical industry is based on ensuring the required product quality while making optimum use of plants, raw materials, and energy. In this context, effective process control using reliable chemical process analytics secures global competitiveness. The setup of those control strategies often originate in process development but need to be transferable along the whole product life cycle. In this series of two contributions, we want to present a combined view on the future of PAT (process analytical technology), which is projected in smart labs (part 1) and smart sensors (part 2). In laboratories and pilot plants, offline chemical analytical methods are frequently used, where inline methods are also used in production. Here, a transferability from process development to the process in operation would be desirable. This can be obtained by establishing PAT methods for production already during process development or scale-up. However, the current PAT (Bakeev 2005, Org Process Res 19:3–62; Simon et al. 2015, Org Process Res Dev 19:3–62) must become more flexible and smarter. This can be achieved by introducing digitalization-based knowledge management, so that knowledge from product development enables and accelerates the integration of PAT. Conversely, knowledge from the production process will also contribute to product and process development. This contribution describes the future role of the laboratory and develops requirements therefrom. In part 2, we examine the future functionality as well as the ingredients of a smart sensor aiming to eventually fuel full PAT functionality—also within process development or scale-up facilities (Eifert et al. 2020, Anal Bioanal Chem).

## Introduction—process analytical laboratories

The knowledge about a traded product is an integral part of its added value. In fact, not only the knowledge about the product itself, but also its entire product life cycle from its raw materials to their return as waste or recyclable material, i.e., the supply chain plays an important role. In that regard, analytical science provides the tools to obtain that knowledge, i.e., information about the materials and their composition. The information content has long since gone far beyond the chemical composition of a product. Many analytical methods also provide information on the spatial and structural distribution of substances or their interactions with each other, which determine their physicochemical properties.

In addition to process control, i.e., optimization of yield, energy input, and quality assurance, process data analysis and process sensor technology provide mandatory information to ensure occupational safety for the acute and sustainable protection of employees and plant safety (protection against deviating and hazardous operating conditions), as well as to guarantee environmental protection and to provide information on plant condition and availability. Without this information, process automation and plant operation would be inconceivable. In many cases, this demands a knowledge-based approach, which significantly contributes to the security of the points mentioned above.

At present, central analytical laboratories have a large scope and depth of chemical analytical competence supporting basic research; process-accompanying analytical laboratories are of utmost importance to the asset life cycle, i.e., process development, scale-up support, and process implementation due to their proximity to the application and the production environment. During production, in-process controls are often carried out in these laboratories because many processes are not yet consistently equipped with online process analytical technologies. The requirements for online chemical analytical equipment for the process industry go far beyond those of other industries. [1, 2] These include in particular explosion protection and hygiene and regulatory requirements as well as requirements for functional safety. In many cases, explicit certification and validation is necessary. These are all tasks which are nowadays performed at the appropriate place by PAT laboratories. However, current requirements go beyond the previous requirements. The prospective features of the PAT labs will allow to fully integrate the real workflows (supply chain and asset life cycle) of the process industry and to support them with cloud-based data acquisition, which collects production data and provides improved models to update production. Analytical data is also collected to support continuous developments from early R&D phases to process analytical technology for optimized production to a much greater extent than today.

In this paper, we first describe the present structure of the process industry in terms of work flows and product and process development. Second, we show where data are generated and how it is integrated into a holistic knowledge management concept—also considering the evolving production concepts and data analysis strategies, which we briefly introduce. From this, we finally derive the requirements for the laboratory and its staff.

### Digitalization of process industry

Industry 4.0 is often associated with the manufacturing industry. However, for the process industry as the third largest and most research-intensive branch of German industry, topics as digitalization, networking, big data, and AI (artificial intelligence) are of considerable importance, too. Both new technical opportunities and new business models are emerging. On the one hand, the digitalization of the process industry, i.e., the manufacturing companies in the chemical, pharmaceutical, life sciences, and food sectors, supports the safe and efficient production of internationally competitive products. On the other hand, it offers manufacturers of equipment and software the opportunity to worldwide export production know-how in the form of measurement and control technology against the background of new tools, methods, and opportunities for networking and intelligent automation. Both contribute to an enormous regional competitive advantage.

**Fig. 1:**
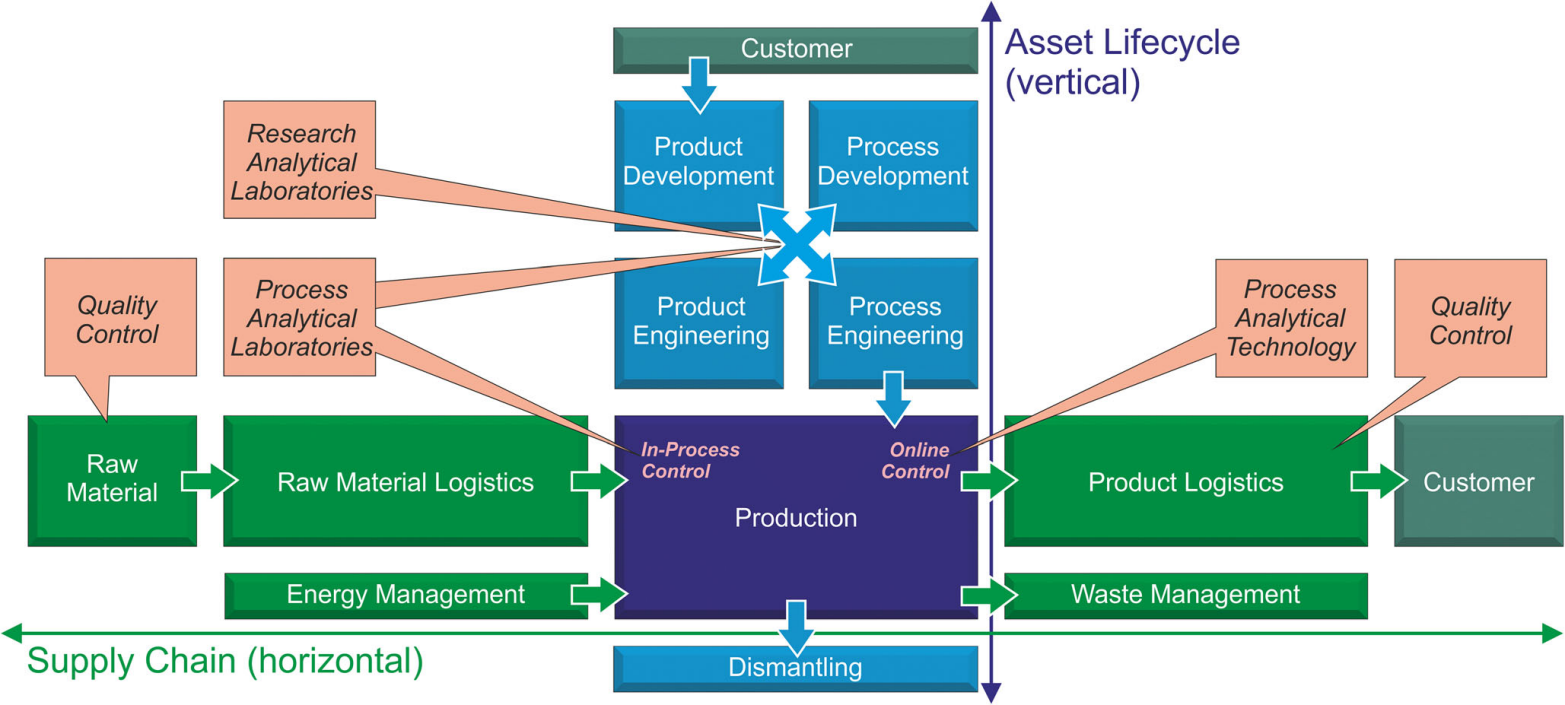
Conventional real-life work flow for process industry: The horizontal supply chain starts on the left with raw materials, which are converted during production to high quality products according to customer specifications (right). The vertical asset life cycle starts at the top with customer needs, which are translated to products and processes by R&D and engineering to an optimized production (bottom). Several chemical analytical laboratories are involved

The process industry is characterized by two supply chains. These are the horizontal supply chain and the vertical asset life cycle of the production plant, both of which come together in the production process (see Fig. 1). The efficiency of the overall system is significantly determined by the fast and comprehensive availability of reliable data from all parts of the two supply chains. For example, a modification of the raw material quality may require adjustments in production, which may already have been investigated in process development and for which information is therefore available. Today, these can only be used “by hand” with considerable difficulty, as the value chains are still sparsely interconnected. The digitalization will bring significant progress here and lead to an increase in process reliability, efficiency, and finally profitability.

With digitalization, process laboratories are enabled to be the basis for a sustainable production. Furthermore, production can be adapted to general requirements and closely linked to both, the asset life cycle and the supply chain. This is referred to as “continuous engineering” (see “Production”).

### Extension of the horizontal supply chain—data collection and analysis

In order to be able to sensibly deal with process and production data that arise within a production cycle, these must first be recorded in a structured manner along all process chains (see Fig. 1). The production cycle begins with the receipt of raw materials: Certificates with the manufacturer’s specifications are available for the raw materials used. As a rule, samples are taken from the raw material and additional chemical analytics are carried out in order to verify the information provided by the manufacturer or to obtain additional information on the composition of the raw materials with a view to release for the intended use. The released goods are usually stored in intermediate warehouses and, if necessary, assembled for production.

### Production

The production process is essentially controlled and monitored by field devices such as pressure, temperature, and flow sensors. To check compliance with specifications, these measurements are partly supplemented by in-process controls (IPCs) or PAT methods, in which samples are taken at specified intervals and analyzed either in the laboratory or close to or during operation with respect to chemical information. If such PAT methods are used, they are not direct-loop and are used for plausibility control today, e.g., as a traffic light function. In contrast, inline measurements with real-time chemical analytics provide a direct feedback for process control. For that purpose, near-infrared or Raman spectroscopy systems with fiber-connected process probes are already very commonly used.

### Release

Once the production process has been completed, chemical analytics are usually carried out to check compliance with the specifications as release test. The product can be delivered, if these requirements are met. If the specification is not met (out of specification, OOS), the product can—if possible—be reused with limited quality features or it must be discarded or refurbished.

### Further processing

After the product has been delivered to the customer or has been put to further use, the production process for this particular product ends.

Many different data sources, structures, and types characterize the data basis in the production environment. The collected data often show outdated structures and implementations being usually not or only to a limited extent networked with each other. Examples are process control systems, which record and process the signals of field devices, shift personnel recording production events in shift books by, often in handwriting, ERP systems (Enterprise Resource Management) managing data from customer or production orders, laboratory databases storing, and evaluating chemical analytics results from quality, release, and in-process controls.

Although this list of examples could be extended, the few ones mentioned already show the complexity of harmonizing and connecting such heterogeneous data streams. Moreover, application-related properties are often not even considered, as these are only collected by the end customer, and are often not or only to a limited extent accessible to production. Although there are systematic differences between continuous and batch production, data collection along process chains already begins with a systematic and uniform designation of substances and materials (e.g., product flows, approaches, partial batches). When analyzing the individual sub-steps of a process, one often finds structures that can be excellently mapped in a relational database. The electronic storage of data is a fundamental prerequisite for this. In most cases, production or laboratory information management systems (PIMS and LIMS) are already available but have so far often been used separately.

### Extension of the vertical asset life cycle—continuous engineering and digital twin

**Fig. 2:**
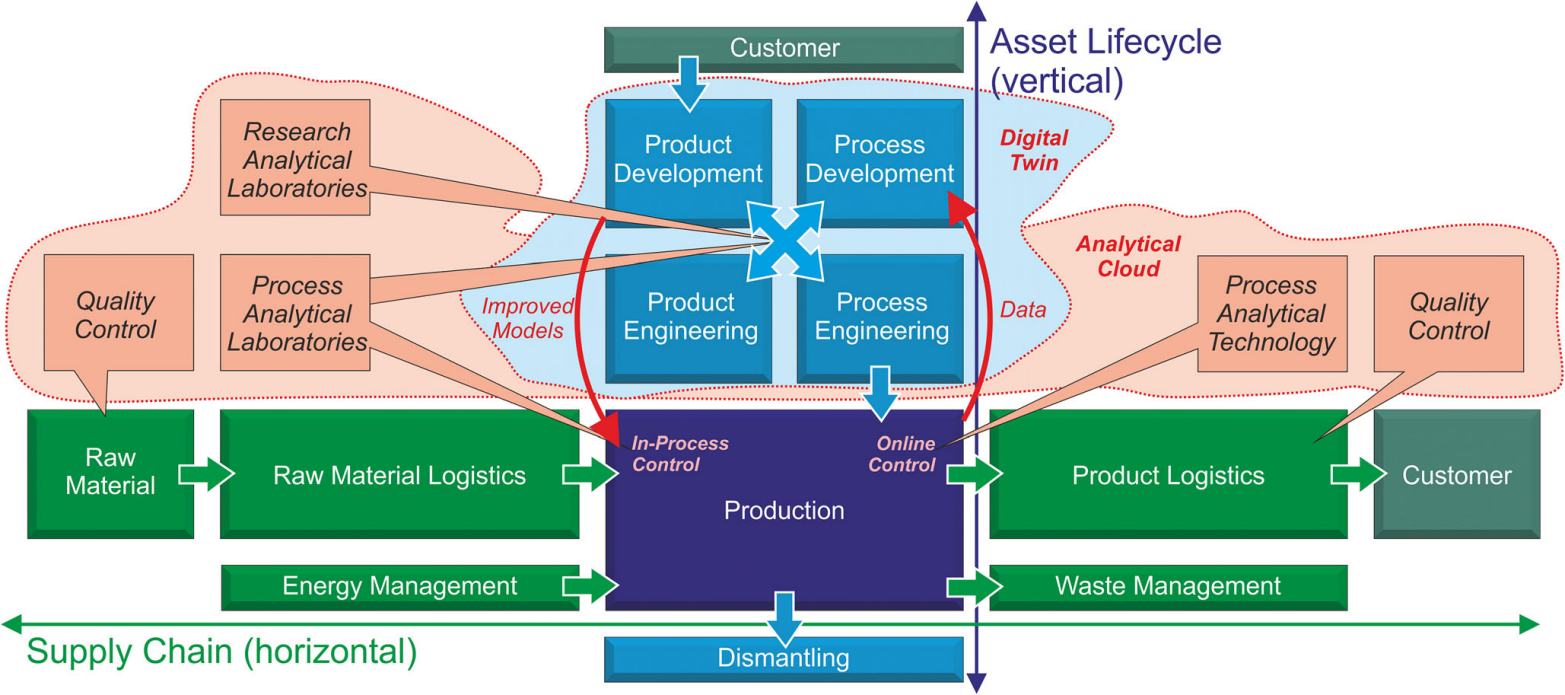
Future real-life work flow for process industry: supply chain and asset life cycle are underpinned by cloud-based data collection. Production data supports digital twins for processes and products while improved models can be used to update the production. Analytical data is collected in a plant-wide cloud to support continuous developments from early R&D phases to process analytical technology for an optimized production

In general, the use of digital plant planning tools makes digital and uniform descriptions of sensors increasingly necessary. Furthermore, their technical data or functionalities must be available in the form of (dynamic) models. This information in turn has to be available to other hierarchical levels in order to simulate a production unit (and later even an entire factory), for example, before it is assembled and operated in its life cycle. An updated and improved digital twin of any individual automation or production component (e.g., with improved models or characteristic curves) can thus be generated from the data collection during the operational process. Through controlled transfer to the operative process, the latter can in turn be optimized (see Fig. 2).

Virtual description supports the required “end-to-end engineering.” The reference architecture model industry 4.0 (RAMI 4.0) [3] and the currently revised IEC 62890 (Life-Cycle Management) very concretely describe both continuous engineering and further dimensions of information exchange. RAMI 4.0 is the key to understand the different hierarchical levels of Industry 4.0. The RAMI model has three dimensions: on the x-axis, the life cycle of the components of a value chain is represented. This begins with a virtual part (“type”), which ends in a dynamic model based on planning. The axis turns into an “instance.” a physical object, which is first assembled and then put to use in the last step, the abovementioned operational process. The y-axis schematically represents different hierarchical levels, ranging from the product/workpiece to the field device level, modules, processes, a company, the enterprise, and the Internet of Things. Finally, different layers of information are represented in the direction of the z-axis. These can include measurement data, status information, and energy states. The individual hierarchical levels are separated from each other and information displayed is only available to authorized users.

Consistent application of this concept is still outstanding today. The generation of a digital twin of an entire production unit or factory still requires several years of development and validation time. For the controlled transfer of the information as a validated model to the operational instances, clearly defined functions and tiered access rights need again to be defined.

### Modular systems reduce complexity through encapsulation

As one of the first manifestations of industry 4.0 and cyber-physical systems, concepts for modular production are derived [4]. Modularization increases the flexibility, availability, and capacity utilization of plants, and different products can be brought to market more quickly. It is therefore also conceivable for module-based specialty chemicals to benefit from a dynamic model of the manufacturing industry and to realize shorter innovation and product cycles. The concept also allows higher quantities of more standardized field devices in modular systems.

In addition to the functional system, it is essential for the success of modularization that descriptions of important system and automation components such as module types, connections, parametrizations, and work flows are available and that these components can be optimally reconfigured for a product into new systems with fast setup times. Each part thus becomes a smart part of networked production. [5]

Modular automation reduces the complexity of engineering, release, commissioning, and maintenance for the user by encapsulating the process engineering functions. The complexity remains but is limited to the inside of the module. The user only sees the interfaces for the coupling of the modules, which “only have to be understood once” through standardization. The understanding of the system reduces the perceived complexity through so-called de-orchestration or decomposition. Currently, two variants are discussed. Variant A: the module is automated by using a small controller to process the required module logics. Only the modules logic runs in the controller and only the required values are exchanged with the controller. Variant B: The modules only use I/O modules and the controller executes the AT logic [3].

## Current role of process analytics laboratories

In contrast to the visions of the future outlined above (see Fig. 1), there is no company-wide monitoring of chemical analytical questions today. Networking at a laboratory level, e.g., communication about experimental tasks, needs, and outputs, and at a data acquisition level, e.g., using cloud systems, are the main challenges for process analytics laboratories today.

Quality assurance laboratories (sample drawing, raw material control, goods’ control for incoming and outgoing goods), R&D laboratories to accompany product development, and analytical laboratories to accompany process development, scale-up, or the implementation of processes are very rarely networked. This also applies to production-related analytical laboratories in which operational analysis and in-process controls are carried out.

In the beginning of the asset life cycle, chemical analytical methods are currently developed without the intention to be implemented in the process later on. Production processes are developed without incorporating a specific “physical-chemical” control method though. As a result, a high proportion of the products are only rejected for release during quality control or in outgoing goods as not complying with the specifications. In such cases, production-accompanying chemical analytics are only subsequently established, which is very often associated with high costs and a negative image on all sides. In-process control work flows are not optimal anyway, as they exert pressure on the employees due to the difficulty of sufficiently good sampling, sample aging, and the time window until the process is readjusted.

Extending chemical analytics with PAT methods would provide enough and further valuable information for the whole life cycle. However, feasibility studies for developing a process analytical technology for an already established process is not trivial, e.g., due to the reproducibility of sampling and sample aging. Therefore, establishing PAT methods straight from the beginning, e.g., already during the process development phase or later during scale-up, would lead to tremendous benefits for the process performance and product quality. Furthermore, a lot would be gained, if all involved laboratories could access a common database and are as close to the users as possible. In addition, there will also be opportunities in the future to make chemical analytical equipment and software more compatible, for example, through standardized and non-proprietary interfaces for control and data processing. This would lead to a better mutual understanding from the respective application-related point of view and thus massively improve the company-wide competencies and the understanding at the interfaces. The different laboratories involved at the several stages of process development and production would still have their specific tasks, but the networking among them would greatly improve their focus and output information and would positively affect the global process quality and efficiency.

Data acquisition using cloud-based systems would significantly support the knowledge gain from process analytical laboratory data. Ideally, all samples and partial samples in a (process) laboratory environment are uniquely coded, e.g., using barcodes, and assigned to the pending examination orders.

As more and more chemical analytical instruments are equipped with contemporary interfaces (e.g., universal, Ethernet-based standards like OPC-UA (open platform communications unified architecture)), there is an alternative to the local evaluation computer: the use of highly available server architecture in decentralized, secure rooms. With the appropriate architecture, even device drivers and evaluation routines run in a validated environment. The failure of hardware can be reliably intercepted via a server network. Each access to the system can be adapted to security requirements by means of user roles and audit trails and if required by monitoring the whole data traffic.

The result files of the examination procedure used contain the sample codes and thus, can be clearly assigned to the samples. Automatic transmission means that neither manipulation nor confusion can occur. Even the device check can be done with the help of the system. Instead of real samples, calibration samples can pass through the system. This always ensures that the system was in a valid state at the time of measurement. Failure to pass equipment checks would result in the blocking of further measurements.

The procedure is confirmed by electronic signature(s). In the regulatory environment, compliance with ISO 17025 as well as the standard under 21 CFR Part 11 (Title 21 of the Code of Federal 20 Regulations, Part 11) is a generally accepted standard [6]. ISPE (International Society for Pharmaceutical Engineering) proposes further standards for automated and software systems, such as GAMP [7].

## Future requirements on smart PAT laboratories

In general, laboratories are central hubs for chemical, biotechnological, pharmaceutical, or food production. They play a key role in research and development, chemical analytics, quality assurance, maintenance, and process control. For process development and optimization, process analytical technology (PAT) has proven to provide powerful tools for improving process understanding, increasing productivity, reducing waste and cost, and decreasing process time.

Those tools together with the concepts of Industry 4.0 will significantly improve effectivity and efficiency. PAT can support process automation, knowledge-based production, and most importantly link single-data streams. Future functionalities of smart sensors—defined in a further contribution [8] as a combination of a chemical analytical device and process intelligence—will allow these sensors to add into the linked data streams. Thus, smart PAT laboratories know what its colleague devices in the field are doing in the production and are able to connect this contextualized information with its own chemical analytics.

### Chemometrics and data analysis

As mentioned above, the specifications of a product are related to many different key parameters from the entire process chain. In addition, although specifications are ultimately based on biological, physical, or chemical parameters or the presence of individual species (main components, by-products, impurities, etc.), they are generally not directly readable from these, but are complexly linked together. Complex mathematical methods can be used to recognize and analyze relationships between properties of the product and the production data. Mathematical tools from the field of statistics, multivariate data analysis, and machine learning include, e.g., principal component analysis, classification, and regression analysis, which in this context are also referred to as chemometric methods [9]. An understanding of the process based on thermodynamic, reaction kinetic, or other process engineering models is todays standard for the optimal design of production plants. In combination with process analytics and sensors and material flow balances, modeling also always provides perfect access to process control in order to ensure optimum product properties in line with the quality-by-design approach.

Chemometric methods are widely used in modern times. Examples of possible applications are in areas such as

- Design of strategies for statistic or chemometric sampling, design of experiments, design of experimental studies,
- Design of protocols for data acquisition (with signal processing), methodologies for data validation and database management (including meta data),
- Quality control, including quality assurance and quality management,
- Data interpretation and data analysis, both when using multivariate (many variables), as well as univariate (one variable) or bivariate (two variables) methods,
- Process control, optimization and monitoring,
- Modeling of chemical processes and properties,
- To manage the decision analysis and to design methods or protocols for decision analysis in process control and optimization
- Validation and calibration of methods and instruments.

Due to the multidisciplinary and interdisciplinary nature of the field of chemometrics, it is often possible to establish unusual links and thus solve problems arising from disciplines as diverse as medical diagnostics, decision sciences, and quality assurance.

### Technology change requires mind change

The interdisciplinary is necessary to get deeper insights in the process itself, but also to provide new ways of production and working. However, the effort to establish and validate new methods or technologies, the associated costs, and possible process modifications lead to long realization times or even hinder it at all. Especially, innovative 4.0 concepts for establishing new data sources, linking, and evaluating data streams require suitable and flexible testing surroundings and new concepts for implementation—for both production, and IT. In that regard, data security and protection are high priority topics throughout the whole implementation process.

Besides, issues arising from a missing “digital mind-set” should not be underestimated. Thus, involving all necessary parties straight from the beginning of a project is essential for the choice, design, and acceptance of a new technology as well as keeping and transferring knowledge within the company. In that regard, scalable projects showing a clear business case, transferring tacit to explicit knowledge as well as appropriate education and training, are keys for successful 4.0-projects.

### Human–machine interfaces and augmented reality

Simplification can also be expected about the human–machine interface. Well-programmed apps, for example, are based on very intuitive and manageable graphical user interfaces and do not require the processing of endless menus or the interpretation of (binary) number sequences. This is also possible without standardization and is based solely on information restriction and justifies the success of the apps in the private sector, while non-harmonized or proprietary menus still make the operation in the industrial sector sometimes very confusing today. Augmented or virtual reality devices can significantly support education and training as well as maintenance or routine tasks. In addition to new requirements on the production and IT infrastructure, suitable technologies shall provide a highly robust and reliable performance throughout its range of application.

Furthermore, technologies should not only be identified and established for isolated applications but also provide knowledge and cross-links along the whole supply chain and asset life cycle. This also means that suitable cross-links along the value chain need to be implemented accordingly to connect new technologies. For example, implementing sensors on a shop level providing modern automation interfaces to higher automation levels without the robustness needed in a chemical plant or the hygiene requirements needed in pharmaceutical production is not useful. In turn, transferring complex data structures on a DCS (distribution control system) or MES (manufacturing execution system) level is not supportive, too. In that case, e.g., edge computing or data pre-processing on a sensor level together with a suitable server or cloud-concept should be considered. The NAMUR open architecture [10, 11] (open process automation) are first small steps to open the classical automation level hierarchy for data collection.

### Joint task: research and equipment/software manufacturers and users

Many projects aiming the development of a smart analytical infrastructure, e.g., by means of smart PAT laboratories, struggle with either infrastructure or technology issues. Current PAT laboratories are focused on providing standardized measurement technology for single chemical analytical tasks often missing both the technology and data cross-link to R&D, process development, quality assurance, maintenance, and IT. Furthermore, tacit knowledge is lost over time making technology improvements and extensions impossible. For example, supporting daily plant tours to check the performance of instrument or construction parts by vibrational sensors or the use of infrared sensors for oil analysis would provide the development of a sustainable predictive maintenance concept. For predictive maintenance, data science tools as machine learning “quantify” the experience of the engineer or technician together with already existing digital information to develop models for predicting maintenance tasks.

**Fig. 3:**
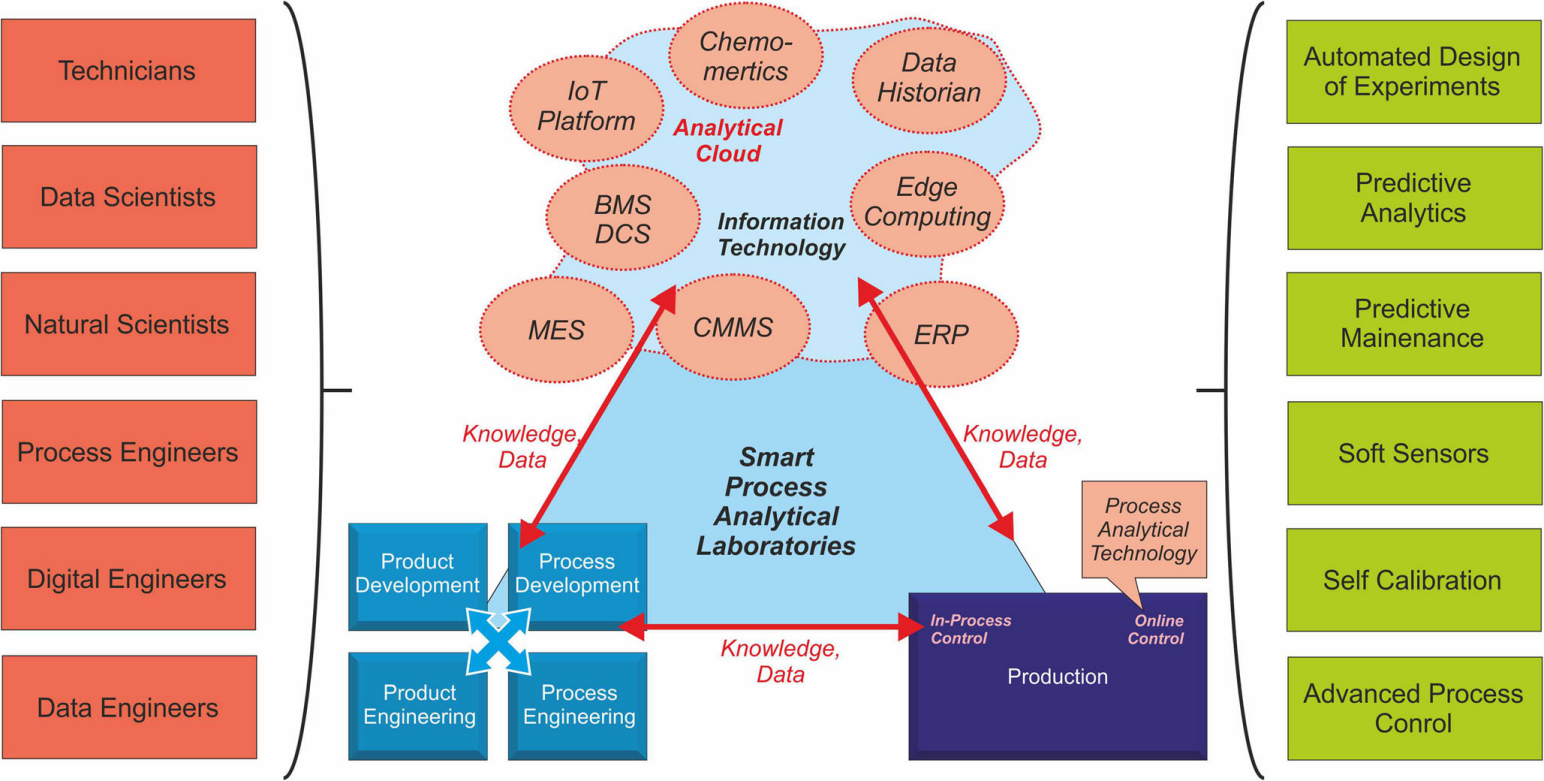
Smart PAT laboratory: the connected PAT laboratory as a central hub to efficiently exchange technology, knowledge, and data between research, shop, and IT level. This will generate new ways of working and technologies for research, development, and production. Thus, it needs collaboration of different disciplines and roles (left) as well as implementation and use of new tools (right)

Technology-wise, this means that IT and measurement knowledge need to be combined in the future as depicted in Fig. 3. Vendors or service hubs need to provide not only the measurement technology but also an appropriate data and IT infrastructure implemented in the company network to collect, and transfer data, and knowledge. Implementing edge computing, IoT platforms, cloud services, or hybrid cloud concepts together with suitable security structures is a solution. However, defining and harmonizing the large variety of data formats and ensure a secure data transfer are the big challenges. Therefore, the close collaborations of data engineers, data scientist, and digital engineers with process engineers, technicians, and natural scientists are of utmost importance (see Fig. 3).

## Conclusion

With the introduction of process analytical technology, innovative digital technologies key process variables can be directly monitored and controlled and thus, securely driven much closer to their limits. Therefore, the process more and more converges to its optimal operating limit. Classical, non-model-based solutions reach their limits when sensor information from several sources must be merged. In addition, their adaptation causes a high effort during the life cycle of the process. This demands adaptive control strategies, which are based on dynamic process models as mentioned above. Model-based control concepts have also the potential to automatically cope with changes of the raw materials as well as process conditions.

Finally, a plant-wide control scheme can be implemented using iterative set-point optimization on basis of local models taking into account the dynamic behavior. When the local controllers are model-based, the response surfaces can be computed from these models. This is not the case if classical control schemes are used, which are based on empirical data.

A comprehensive infrastructure for chemical analytics along with powerful data analysis and model maintenance will help to optimize both assets and systems. Predictive analytics will be installed to reduce unplanned down-times. Newly available information generated by these tools will lead to new, transformative business models supported by new applications. Instead of offering physical products for sale, companies will increasingly offer products as a service.

## Summary

The consistent use of process analytics together with smart digital tools leads to an increase in process and plant safety. The data gained together with suitable information management systems increases the knowledge about the process and thus leads to preventive assurance of the required quality in the sense of a specification-compliant product.

Smart process laboratories as described here together with holistic knowledge management pave the way for most flexible future processes. Inversely, such digitalization-based knowledge management will also contribute to faster product and process development.

Ultimately, the increase in confidence in these complex technologies must not be neglected, but seen as a joint task of research, equipment, and software manufacturers together with the users.

The abovementioned potentials of PAT are even amplified when combined with smart sensors, which scope is elaborated in part 2 of this series.
